# Comparison of Thermal Response for RF Exposure in Human and Rat Models

**DOI:** 10.3390/ijerph15102320

**Published:** 2018-10-22

**Authors:** Sachiko Kodera, Akimasa Hirata

**Affiliations:** Department of Electrical and Mechanical Engineering, Nagoya Institute of Technology, Nagoya 466-8555, Japan; ahirata@nitech.ac.jp

**Keywords:** human safety, thermal time constant, temperature elevation, vasodilation, bioheat transfer equation

## Abstract

In the international guidelines/standards for human protection against electromagnetic fields, the specific absorption rate (SAR) is used as a metric for radio-frequency field exposure. For radio-frequency near-field exposure, the peak value of the SAR averaged over 10 g of tissue is treated as a surrogate of the local temperature elevation for frequencies up to 3–10 GHz. The limit of 10-g SAR is derived by extrapolating the thermal damage in animal experiments. However, no reports discussed the difference between the time constant of temperature elevation in small animals and humans for local exposure. This study computationally estimated the thermal time constants of temperature elevation in human head and rat models exposed to dipole antennas at 3–10 GHz. The peak temperature elevation in the human brain was lower than that in the rat model, mainly because of difference in depth from the scalp. Consequently, the thermal time constant of the rat brain was smaller than that of the human brain. Additionally, the thermal time constant in human skin decreased with increasing frequency, which was mainly characterized by the effective SAR volume, whereas it was almost frequency-independent in the human brain. These findings should be helpful for extrapolating animal studies to humans.

## 1. Introduction

The rapid development of wireless technology has changed the electromagnetic environment, leading to concerns regarding potential adverse health effects caused by electromagnetic field exposure. In radio frequency (RF) exposure (above 100 kHz), the dominant factor is the thermal effect. Therefore, induced temperature elevation in biological bodies is an important factor. There are two international guidelines/standards for human protection from electromagnetic fields, which have been mentioned by the World Health Organization (WHO) as international recommendations: the International Commission on Non-ionizing Radiation Protection (ICNIRP) guidelines [1] and IEEE standards [2].

In the guidelines/standards, the metric for human protection against RF fields is the specific absorption rate (SAR) averaged over 10 g of tissue for RF-localized fields from 100 kHz to 3 GHz [2] or to 10 GHz [1]. At frequencies higher than 3 or 10 GHz, surface heating becomes dominant; therefore, incident power density is used as a metric for human protection instead of SAR. The SAR limit is 10 W/kg for occupational exposure or in restricted areas, and a reduction factor of five is applied for the general public or in unrestricted areas. Wireless devices used in our daily life are designed to comply with the SAR limit for general public (2 W/kg). 

The rationale for the limit is based on studies of thermal damage in animals [3,4], which were then extrapolated to humans (see the review in ICNIRP [5] and IEEE standards [2]). This was partly because of ethical reasons. To the best of our knowledge, only the authors’ group has developed a computational code for temperature computation in animals, taking into account their thermoregulation [6,7]. Even for humans, only a few groups have succeeded in this [8,9,10,11]. Clarifying the difference of temperature elevation and its time evolution between humans and animals may contribute to the scientific rationale for establishing international guidelines/standards. 

In the guidelines/standards, the SAR should be averaged over a 6-min period for frequencies lower than 3 GHz [2] and 10 GHz [1]. The rationale behind the averaging time is not clearly mentioned in the guidelines/standards; according to [12], this time was originally “0.1 h”, which implies lower precision than 6 min. Thermal damage, for example, occurs when rabbit eyes are exposed to high-intensity RF waves for ≥30 min [13,14]. Moreover, many studies examined thermal damage in rats [15,16,17]. The WHO workshop in 2002 [18], ICNIRP thermal damage workshop [19], and United States Food and Drug Administration (FDA) thermal damage workshop concluded that thermal limits should consider both temperature and time of exposure whenever possible. Foster et al. [12] derived an analytic formula to estimate the thermal time constant using a one-dimensional homogeneous model for intense brief exposures, especially at millimeter waves. Some studies using human anatomical models computed the thermal time constants for handset antennas without considering thermoregulation [20,21,22,23]. However, no reports have compared thermal time constants between humans and animals, making direct extrapolation of findings in animals to humans difficult. Note that thermal damage depends on the time–temperature elevation relationship [24], which is not considered in the international guidelines/standards. This is because the limits for the SAR and incident power density are set so that the allowable temperature elevation is well below the point of thermal damage.

We computed the time courses of temperature elevation, including the thermal time constants, in rat and human head models for several exposure scenarios. In our previous studies, an integrated computational technique, combining electromagnetics and thermodynamics, was developed by incorporating a thermoregulatory response model based on measured values [7,25]. This study aims to clarify the difference of the temperature elevation and its time course between rats and humans exposed to local RF fields, which is important to estimate how conservative the current limits are for local exposure in the international guidelines/standards.

## 2. Models and Methods

### 2.1. Computational Rat and Human Models

One rat and four human models were considered as computational models. The computational rat model (8-week-old rat) was developed from computed tomography (CT) images [7]. The rat model was composed of the following six tissues: muscle, skin, fat, eye, bone, and brain. These tissues were classified in a semi-automatic procedure and then additional partial manual editing by an expert using a similar method to [26] was performed. This model had a resolution of 0.25 mm, and the whole-body weight used in the rat model corresponded to 265 g.

Realistic anatomical human models for different ages and gender were considered. The Japanese male model (TARO) [27], and the European models of the Virtual Family (Duke, Billie, and Ella) [28] were used. These computational models had a resolution of 0.5–2 mm. They were segmented into 51–77 anatomical regions, such as skin, muscle, and bone. The human models were truncated at the bottom of the neck because the rest of the body marginally affects the interaction with the dipole antenna [29]. A major source of computational error in the finite-difference time-domain (FDTD) method is the discretization error, which depends on the ratio between the cell size and the wavelength in biological tissue. A rule to suppress the numerical dispersion error in FDTD simulations is that the maximum cell size should be smaller than one-tenth of the wavelength [30]. Thus, the model resolution was adjusted to 0.5 mm to ensure computational accuracy.

### 2.2. Computational Models

The SARs in the models were first computed using an FDTD method for electromagnetics. The time course of the temperature elevation was then calculated by solving Pennes’s bioheat transfer equation (BHTE) considering the vasodilation. The bioheat modeling has been fully described in our previous studies and is thus reviewed briefly here. 

#### 2.2.1. SAR Computation

The FDTD method was used to compute the RF power absorbed in the computational human head and rat models [30]. Convolutional perfectly matched layers (CPML) were used as the absorbing boundary conditions for absorbing outgoing scattered waves to simulate an infinite space [31]. The dielectric properties of each type of tissue were determined with a Cole–Cole dispersion model in both rat and human models [32]. The variability caused by different sets of dielectric properties has been shown to be marginal [33]. The SAR was defined for sinusoidal waves as follows:

(1) $$SAR\left(r\right)=\frac{\sigma \left(r\right)}{2\rho \left(r\right)}\;{\left|E\left(r\right)\right|}^{2},$$

where |**E**| denotes the temporal peak value of the electric field at position **r**, and the parameters *σ* and *ρ* denote the conductivity and mass density of the tissue, respectively. 

The SAR was averaged over 10 g of tissue in a cubic shape following the IEEE standard [34]. Even though the SAR is not used at frequencies above 6 GHz by the IEEE standard [2], the same metric was used at 10 GHz for a proper comparison. The 10-g SAR averaged in a single contiguous tissue following the ICNIRP guidelines [1] was difficult to use in the rat model, because the rat skin weight over the whole body was 32 g. 

#### 2.2.2. Temperature Computation

The BHTE considers heat exchange mechanisms, including heat conduction, blood perfusion, and resistive heating, and was shown as follows [35]:

(2) $$\begin{array}{c} C\left(r\right)\rho \left(r\right)\frac{\partial T\left(r,t\right)}{\partial t}=\nabla \left(K\left(r\right)\nabla T\left(r,t\right)\right)+\rho \left(r\right)SAR\left(r\right)\\ +M\left(r\right)-B\left(r,t\right)(T\left(r,t\right)-T_{B}), \end{array}$$

where **r** and *t* denote the position vectors in tissue and the time, respectively, terms *T* and *T_B_* denote the temperatures of tissue and blood, respectively, the term *C* denotes the specific heat of the tissue, *K* denotes the thermal conductivity of the tissue, *M* denotes the metabolic heat, and *B* denotes the factor related to the blood perfusion. The SAR obtained from Equation (1) was substituted into Equation (2) as a heat load. While the electromagnetic wave instantaneously reaches steady state, the time constant of the heat takes several minutes at superficial tissues [12], and over 30 min at deep tissues [36]; the time variation of SAR is much smaller than the thermal constant and thus can be assumed as a constant during exposure.

The boundary condition between tissues and internal/external air is as follows:

(3) $$-K\left(r\right)\frac{\partial T\left(r,t\right)}{\partial n}=H\left(T\left(r,t\right)-T_{a}\right),$$

where *T_a_*, *H*, and *n* denote the ambient temperature, heat transfer coefficient, and vector normal to the body surface, respectively. The initial temperature *T*_0_ was found using Equations (2) and (3) for SAR = 0 W/kg, *T_B_*_0_ = 37 °C, and *T_a_* = 27 °C.

The blood temperature in the rat model changed to satisfy the first law of thermodynamics as follows [8,37]:

(4) $$T_{B}\left(t\right)=\int \frac{Q_{BT}\left(t\right)-Q_{BT}\left(0\right)}{C_{B}\rho_{B}V_{B}}dt,$$

(5) $$Q_{BT}\left(t\right)=\int B\left(t\right)\left(T_{B}\left(t\right)-T\left(r,t\right)\right)dV,$$

where *Q_BT_* denotes the total heat quantity of tissue and blood, and terms *C_B_* (=4000 J/kg/°C), *ρ_B_* (=1058 kg/m^3^), *T_B_*_0_, and *V_B_* denote the specific heat of blood, mass density of blood, initial blood temperature, and total volume of blood, respectively. The average blood volume per unit of rat body mass was considered to be 64 mL/kg [38]. The blood volume of the rat model was set to 18.9 mL. The blood temperature in the human model was fixed at 37 °C and sweating was ignored, because it marginally affects the temperature elevation for local RF exposure [29].

Skin blood perfusion was expressed as follows, based on the cutaneous veins of a dog with local warming [39]:

(6) $$B\left(r,t\right)=\left\{B_{0}\left(r\right)+F_{HS}\Delta T_{H}\left(t\right)+F_{SS}\Delta T_{S}\left(t\right)\right\}\cdot 2^{\Delta T\left(r\right)/6},$$

where *B*_0_ denotes the basal blood perfusion of each tissue, ∆*T_S_*(*t*) denotes the average temperature elevation of the skin, and ∆*T_H_*(*t*) denotes the elevation of the core temperature. Additionally, *F_HS_* (=17,500 W/m^3^/°C) and *F_SS_* (=1100 W/m^3^/°C) are the coefficients that determine the changes in the blood perfusion characteristics over time [40]. Thus, the blood and hypothalamus temperature elevations were used as the approximate body core temperature in rat and human models, respectively.

The blood perfusion in the brain was given as follows, based on our rat measurements for local RF exposure [7]:

(7) $$B\left(r,t\right)=B_{0}\left(r\right)\left(1+F_{HS}\Delta T_{H}\left(t\right)\right)\cdot 2^{\Delta T\left(r\right)/F_{BB}},$$

where *F_HB_* (=0.053 °C^−1^) and *F_BB_* (=13.9 °C) denote weighting coefficients in relation to the variations in the core and brain temperature elevations, respectively [25]. 

Vasodilation in tissues, except for the skin and brain, was given as follows [41,42]:

(8) $$\begin{array}{c} B\left(r,t\right)=B_{0}\left(r\right),\;T\left(r,t\right)\leq 39{\;}^{{}^{\circ}}C\\ B\left(r,t\right)=B_{0}\left(r\right)\left\{1+S_{B}\left(T\left(r,t\right)-39.0\right)\right\},\;39{\;}^{{}^{\circ}}C\leq T\left(r,t\right)\leq 44{\;}^{{}^{\circ}}C\\ B\left(r,t\right)=B_{0}\left(r\right)\left\{1+5S_{B}\right\},\;44{\;}^{{}^{\circ}}C\leq T\left(r,t\right) \end{array}$$

where *B*_0_(*r*) is based on the blood perfusion in each tissue and *S_B_* (=0.8 °C^−1^) denotes a coefficient that determines the change in the blood perfusion characteristics over time.

The thermal parameters of the rat and human models were taken from [25,43], respectively. The heat transfer coefficient *H* was set to 0.5 W/(m^2^·°C) between the skin and air, and 8.1 W/(m^2^·°C) between lung and inner air in the rat model [7]. The heat transfer coefficient *H* was set to 5 W/(m^2^·°C) between the skin and air [44], and 20 W/(m^2^·°C) between the eye and air [45] in the human model. Sweating by the human was not considered, as temperature elevation is confined around the surface in most cases, which is not sufficient to induce sweating [29,46]. Variability of different modeling of the temperature elevation can be found in [47]; typically, the blood perfusion rate is the dominant parameter affecting local temperature elevation.

#### 2.2.3. Exposure Scenarios

**Figure 1:**
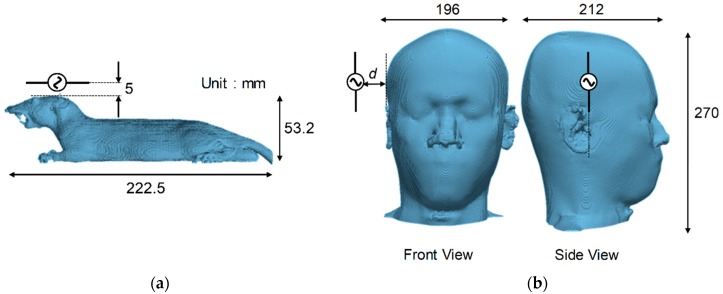
Exposure scenarios for computational (**a**) rat and (**b**) human head models (TARO). The separations between the antenna and the rat and human models are 5 mm and 5–25 mm, respectively.

Figure 1 illustrates the exposure scenarios. As shown in Figure 1, the separation between the dipole antenna and the surface of the head model was 5 mm for the rat, and 5, 15, and 25 mm for the human. The frequencies considered here were 3, 6, and 10 GHz, which are the upper frequency range at which the SAR is used. The reason for choosing frequencies above 3 GHz is that the whole-body-averaged SAR cannot be neglected at lower frequencies, making it difficult to discuss the local temperature elevation and its time course. The antenna–head separation for the rat model was only chosen at 5 mm to realize local exposures [7,17,25]. The length of the antenna was adjusted to half the wavelength of each frequency in free space. The duration of the RF exposure corresponded to 50 min so that temperature elevation was in the steady state condition. For comparison, the output power of the antenna was adjusted so that the peak SARs averaged over 10-g tissue were 2, 10, 20, 30, 40, and 50 W/kg. The guideline/standards were set at 2-W/kg local RF exposure limit for the general public and unrestricted environments, and at a 10-W/kg limit for restricted environments or occupational exposure. 

#### 2.2.4. Definition of Thermal Time Constant

The thermal time constant is an empirical quantity that characterizes the time needed to reach steady-state temperature (the step response of the tissue). A small thermal time constant indicates a fast thermal response. The thermal time constant was calculated from the time evolution of temperature from continuous wave exposure as:

(9) $$\Delta T\left(t\right)=\Delta T_{max}\left(1-e^{-\left(t-\tau_{D}\right)/\tau }\right),$$

where Δ*T_max_* (°C) is the maximum temperature elevation, *τ* (s) is the thermal time constant, and *τ_D_* (s) is the heat conduction time from the model surface.

The time evolution of temperature was observed as a primary delay function in surface tissues; Δ*T*(*t*) = Δ*T_max_* (1 − e^−*t*/*τ*^) can be obtained by substituting *τ_D_* = 0 in Equation (9). The temperature elevation at the thermal time constant is approximately 63% of the maximum temperature elevation, because Δ*T*(*τ*) = Δ*T_max_* (1 − 1/*e*) ≈ 0.63*T_max_* when *t* = *τ*. The twice and three times values of the thermal time constant approximately correspond to temperature elevations of 86% and 95%, which may be more relevant to human safety, as discussed in Appendix A of the ICNIRP draft guidelines [48]. 

A secondary delay function, *τ_D_* ≠ 0 in Equation (9), was observed in inner tissue (such as the brain). The heat conduction time was observed as the time taken for the heat source (the RF power absorption) to reach the observation point. The observation points of the thermal time constant were taken as the points of the maximum temperature elevation in the brain and tissues (excluding the pinna). Here, the brain tissue included gray matter, white matter, cerebellum, thalamus, pineal gland, and hypothalamus.

#### 2.2.5. Definition of Effective SAR volume

The temperature elevation is mainly affected by the SAR value when larger than 1/*e* of peak SAR [49]. Hirata et al. estimated the heat diffusion length in terms of the length, where the amplitude of heat decreases to 1/*e* [43,50]. Foster et al. derived an analytic formula to estimate the thermal time constant in a one-dimensional homogeneous model for plane wave exposure [49]. Hashimoto et al. proposed the effective area of the SAR pattern as an evaluation index of temperature elevation, which is defined by the area where SAR is larger than 1/*e* of the peak value in the averaging area using a multilayer cubic model [51]. The effective SAR volume *V_eff_* [cm^3^] is defined as a metric where SAR is larger than 1/*e* of the *SAR_max_* to consider both the depth and the spread on the model surface. Here, *SAR_max_* was treated as a value excluding the SAR voxels, and had the highest SAR value of 0.01% (see Appendix A).

## 3. Computational Results

### 3.1. Distribution of SAR and Temperature Elevation

**Figure 2:**
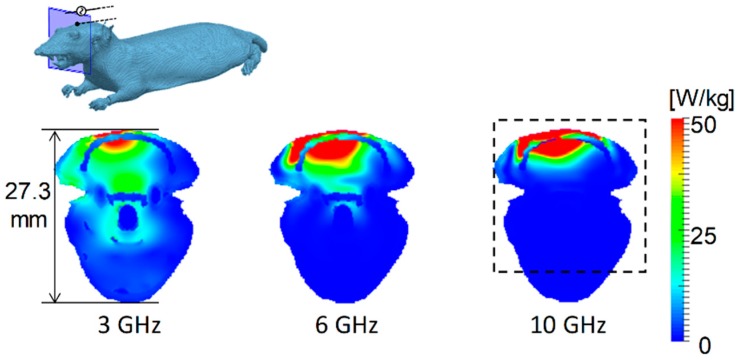
Specific absorption rate (SAR) distributions in the rat model at 3, 6, and 10 GHz. The distance between the skin surface and the dipole antenna was set to 5 mm. The output power of the antenna was adjusted so that the peak SAR averaged over 10-g tissue was equal to 10 W/kg. The dashed square shows the region with a computed peak of 10-g SAR.

Figure 2 shows the SAR distribution on the cross section of the rat through the antenna feed point at 3, 6, and 10 GHz. The peak SAR averaged over 10 g of tissue was adjusted to 10 W/kg for proper comparison. As shown in Figure 2, the SAR was distributed over the entire head at 3 GHz, whereas it is more localized at 10 GHz. Because of the smaller dimensions of the rat head, SAR averaged over 10 g of tissue almost coincides with the average SAR of the entire head.

**Figure 3:**
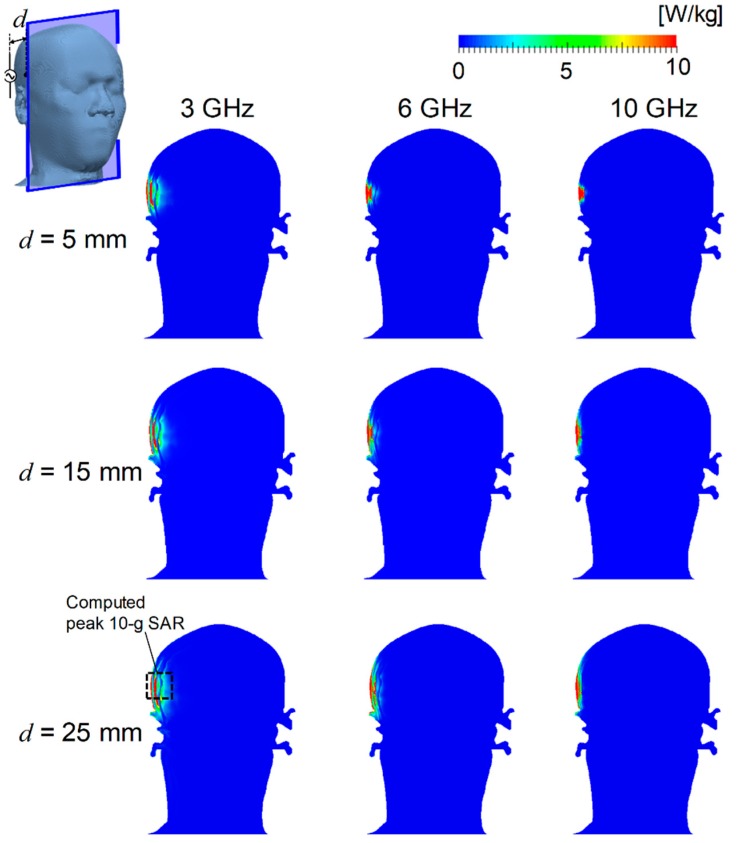
SAR distribution in the TARO model at 3, 6, and 10 GHz. The distances between the skin surface and the dipole antenna *d* were set to 5, 15, and 25 mm. The output power of the antenna was adjusted so that the peak SAR averaged over 10-g tissue was equal to 10 W/kg. The dashed square shows the region with a computed peak of 10-g SAR.

Figure 3 shows the SAR distributions on the cross section of TARO through the feed point at 3, 6, and 10 GHz. The distances between the antenna and surface of the head were 5, 15, and 25 mm at each frequency. The peak SAR averaged over 10-g tissue was adjusted to 10 W/kg, as for the rat model. The SAR was also distributed in the brain surface at 3 GHz, whereas for frequencies above 6 GHz, it was only distributed in the superficial tissues (such as skin and muscle), because of shallower penetration depth of the electromagnetic fields. Further, the lateral extent of SAR was more widespread at lower frequencies. As shown in Figure 3, the exposure volume was smaller for the antenna distance *d* = 5 mm than that at *d* = 25 mm. 

**Figure 4:**
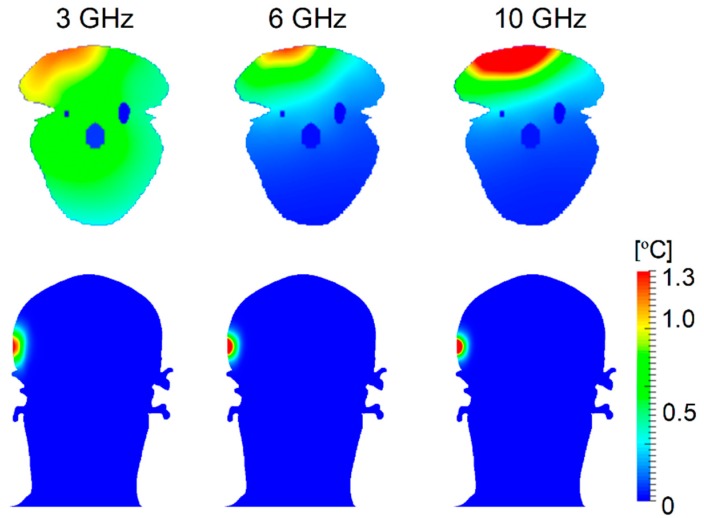
Distribution of temperature elevation in the rat and TARO models at 3, 6, and 10 GHz. The distance between the skin surface and the dipole antenna was set to 5 mm. The output power of the antenna was adjusted so that the peak SAR averaged over 10-g tissue was equal to 10 W/kg.

Figure 4 shows the temperature elevation distributions in the rat and TARO at 3, 6, and 10 GHz. The distance from the head to the antenna was 5 mm. The temperature elevation had a similar tendency to SAR, but was smoother owing to heat diffusion.

### 3.2. Comparison of Temperature Elevation in Rat and Human Models

**Figure 5:**
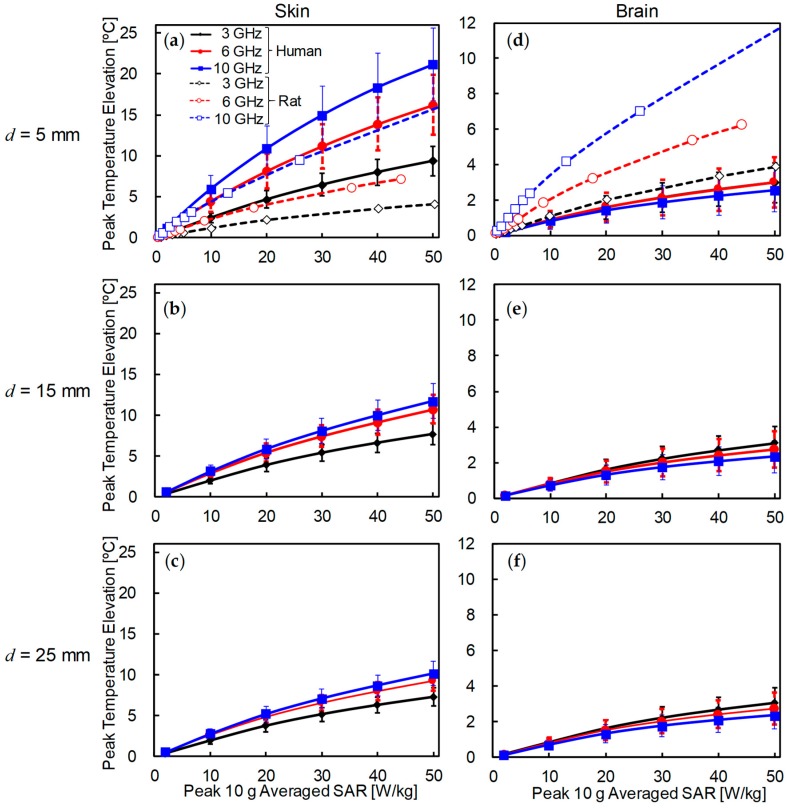
Peak temperature elevation of (**a**–**c**) skin and (**d**–**f**) brain for each peak SAR averaged over 10-g tissue. The distances between the skin surface and the dipole antenna *d* were set to (**a**,**d**) 5 mm, (**b**,**e**) 15 mm, and (**c**,**f**) 25 mm. Each point represents the average value and error bar showing the standard deviation (*n* = 4). For comparison, the temperature elevation of rat brain tissue was also plotted.

Figure 5 shows the peak temperature elevation of skin and brain in the human models for different SARs and antenna–head distances. For comparison, the temperature elevation in the rat skin for *d* = 5 mm was also plotted. As shown in Figure 5a–c, the temperature elevation in the skin increased as the frequency increased. The peak temperature elevation in the human model was approximately twice as high as that in the rat at *d* = 5 mm. The peak temperature elevation decreased as the antenna was moved away for the human. For *d* = 15 and 25 mm, the temperature elevation in the human model was lower than that in the rat model at 10 GHz. As shown in Figure 5d–f, the peak temperature in the brain varied significantly with frequency for the rat model, but was almost independent of frequency for the human model. The peak temperature elevation in the human brain was lower than that in the rat brain at *d* = 5 mm.

### 3.3. Thermal Time Constant in Rat and Human Head Models

**Figure 6:**
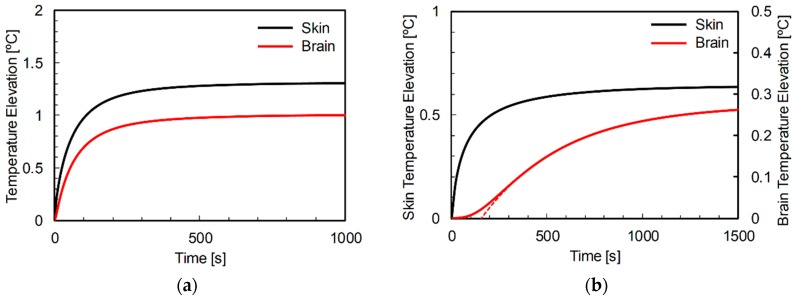
Time evolution of temperature elevation in the (**a**) rat and (**b**) TARO models at 10 GHz. The SAR averaged over 10-g tissue was adjusted to 2 W/kg. The distances from the antenna to the head were (**a**) 5 mm and (**b**) 25 mm. The dotted line in (**b**) shows the regression curve from Equation (9).

Figure 6 shows typical examples of the time evolution of temperature elevation in the rat and human models. The frequency was chosen as 10 GHz, and TARO was used for the human model. As shown in Figure 6a, the heat conduction time (*τ_D_* in Equation (9)) was not necessary to describe the temperature elevation in the skin of rat or human. The time evolution of temperature in the skin was expressed as a primary delay function. As shown in Figure 6b, the heat conduction time in the rat brain was marginal, whereas that in the human brain was significant. The thermal time constant in the human brain was expressed as *τ* + *τ_D_* for comparison with the rat in the following discussion.

**Figure 7:**
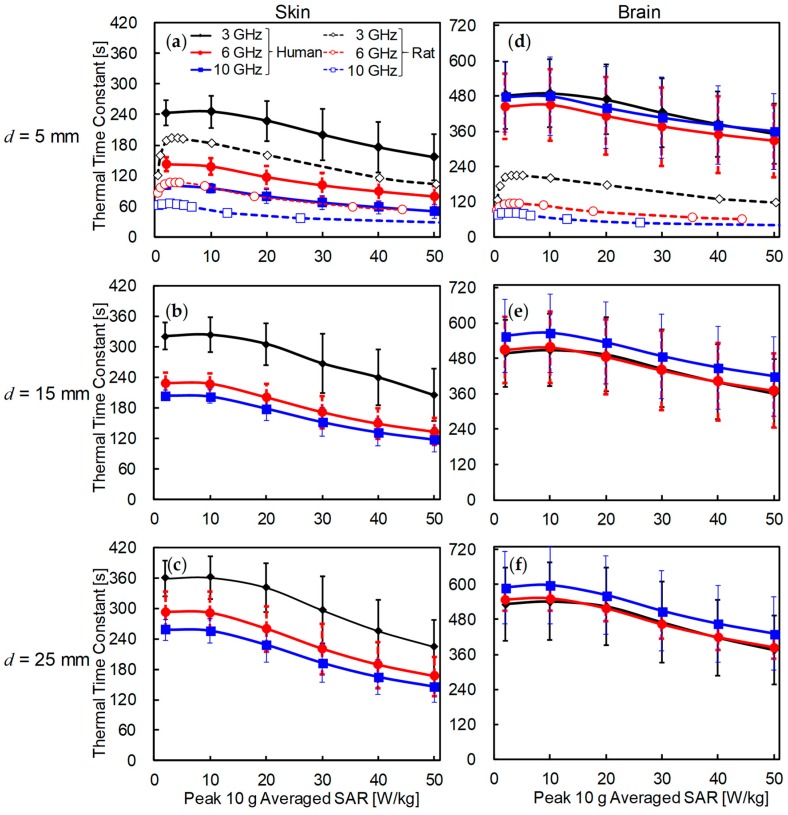
Thermal time constants of (**a**–**c**) skin (*τ* in Equation (9)) and (**d**–**f**) brain (*τ + τ_D_* in Equation (9)) in human models for each peak SAR averaged over 10-g tissue. The distances between the skin surface and the dipole antenna *d* were set to (**a**,**d**) 5, (**b**,**e**) 15, and (**c**,**f**) 25 mm. Each point represents the average value and error bar showing the standard deviation (*n* = 4). For comparison, the temperature elevation of rat brain tissue was also plotted at the 5-mm distance.

Figure 7 displays a comparison of the thermal time constant of the skin and brain in the rat and human models. The thermal time constant was computed at the positions where the peak temperature elevation appeared. The thermal time constant nonlinearly decreased with increasing exposure intensity. As shown in Figure 7a–c, the thermal time constant of the skin decreased with increasing frequency in both rat and human models. The thermal time constant also increased with increasing distance between the antenna and the skin surface. The thermal time constant in the human skin was 1.5 times higher than that in the rat skin at *d* = 5 mm. 

The thermal time constants of skin in the rat and human models were 3 and 6 min, respectively. The time required to reach the thermal steady state was estimated as 6 and 12 min in rat and human models, respectively (2*τ* in Equation (9), which is the time to reach 86% of steady-state temperature). 

As shown in Figure 7d–f, the thermal time constant in the rat brain had similar tendencies to rat skin. One noteworthy point is that the time constant in the human brain was almost frequency-independent. The thermal time constant in the brain of the human was approximately two to four times larger than that in the rat brain at the same antenna–head distance. The maximum thermal time constant of rat brain tissue was 3.5 min at 3 GHz, and that of the human brain was 10 min at 10 GHz for *d* = 25 mm. The time required to reach the thermal steady state was 7 min and 20 min (or more) in rat and human models, respectively.

### 3.4. Relationship between Effective SAR Volume and Thermal Time Constant

**Figure 8:**
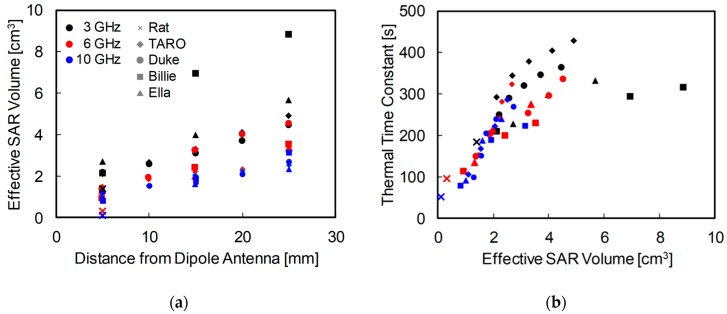
(**a**) Effective SAR volume for each exposure scenario; and (**b**) relationship between the thermal time constant of skin and effective SAR volume in rat and human models. The output power of the antenna was adjusted so that the peak SAR averaged over 10-g tissue was 10 W/kg.

Figure 8 shows the relationship between the effective SAR volume and thermal time constants of the skin at the same intensity of RF exposure in rat and human models. Figure 8a shows the effective SAR volume for each exposure scenario. The effective exposure volume increased with decreasing frequency and increasing antenna–head separation. Figure 8b shows the relationship between the thermal time constant in the skin and the effective SAR volume. As shown in Figure 8b, the thermal time constant depended on the head shape rather than on the frequency, especially at frequencies of 6 and 10 GHz. As expected, the thermal time constant increased as the effective SAR volume increased in all models. 

## 4. Discussion

The thermal time constant has not been discussed until recently [23], except for a few studies on the exposure from handset antennas [20,21,22,23]. Instead, thermal damage in animal studies has been commented on in the international guidelines/standards. For example, regarding the eye exposure in rabbits, temperature elevation was observed in the eye and the core, and then cataract formation was reported [13,14]. To understand the phenomena as well as future design of experiments, the time course of the temperature elevation in the animal is important [13]. Thus, this study computed the temperature elevation in human and rat models for exposure at 3–10 GHz, taking into account the thermoregulation (vasodilatation). In addition to the temperature elevation, thermal time constants were evaluated and discussed.

We demonstrated the difference between SAR and temperature distributions in the rat and human models. The ratios of local- and whole-body-averaged SAR were 18, 26, and 27, at 3, 6, and 10 GHz in the rat model, respectively. The SAR was distributed over the whole head at 3 GHz and in the rat brain, even at 10 GHz (Figure 2). The penetration depth of the skin is 18.8 mm at 3 GHz, 8.2 mm at 6 GHz, and 3.8 mm at 10 GHz [52]. The distance between the brain and the head surface was at most 2 mm in the rat. In the human model, the SAR was marginally distributed in the brain only at 3 GHz (Figure 3). The distance between the brain and the head surface of TARO was 13.5 mm, which was larger than the penetration depth above 6 GHz. As shown in Figure 4, the temperature elevation was distributed throughout the brain in the rat model but was contained locally in the human model. The temperature elevation above 0.1 °C was reached at approximately 2 cm in depth in the human model. 

We then compared the temperature elevation in rat and human models for different antenna distances and SARs. As shown in Figure 5, the peak temperature elevation in human skin was twice that of rat skin at the same head–antenna distance. This difference is attributable to the SAR distribution (volume of power absorption). The temperature elevation was characterized by the heat diffusion length in the volume (approximately 1 cm [50]). In addition, the SAR averaged over 10-g tissue in cubic shape was used as an index of comparison. Further, in the rat model, the skin is close to the brain, where blood perfusion is high, thus reducing skin temperature. In the brain, the peak temperature elevation in the human model was lower than in the rat model. Unlike the rat, the SAR was only slightly distributed in the human brain, and temperature elevation by heat conduction from external tissues became dominant. 

We compared the thermal time constants of rat and human models. As shown in Figure 7, the thermal time constant decreased at higher exposure intensity owing to the cooling effect of vasodilation or nonlinear thermoregulation. The thermal time constant also increased with the increase of the antenna–head distance, which can be attributed to a larger effective SAR volume. The effective SAR volume increased with the head–antenna distance, and may be related to the head size, which has been extensively discussed in [53,54]. The thermal time constant in skin exhibited good correlation with the effective SAR volume, even if the head shapes were significantly different, such as in rats and humans. As shown in Figure 8, the thermal time constant increased as the effective SAR volume increased, but did so in nonlinear fashion. Focusing on each condition, the thermal time constant can be expected to saturate as the effective SAR volume increases. The increment of the effective SAR volume results in thermal diffusion, causing the delay in temperature elevation [23]. This result suggests that the effective SAR volume can be estimated as a metric in superficial tissues. Note that the SAR volume is not a good metric for 5 cm^3^ or less, because it is larger than the heat diffusion length. The difference of the time constant in skin between rat and human models is caused by the difference of the effective SAR volumes (see Figure 8). The effective SAR is useful for estimating the thermal time constant of superficial tissue, which varies greatly depending on the shape and size of the head, type and position of antenna, and the frequency applied.

In the inner tissue, such as the brain, the thermal time constant is also characterized by the heat conduction time, which is mainly attributable to the distance from the heat source to the target point. The heat conduction time (*τ_D_* in Equation (9)) observed in the human brain was approximately 40, 120, and 150 s at 3, 6 and 10 GHz, respectively. In contrast, *τ* decreases as the frequency increases. Therefore, the thermal time constant (*τ + τ_D_*) in the human brain is less sensitive to frequency. The difference of the time constant between rat and human brains is caused by the heat conduction time and differences in temperature elevations.

The limitation of this study is in the modeling of the thermoregulatory response. We used the vasodilation models of the brain and skin that were based on our rat measurements for local RF exposure [25] and cutaneous veins of a dog with local warming [39], respectively. The thermoregulatory response model for blood perfusion was assumed to be the same for both rat and human because of the lack of specific data. In our earlier studies, the thermoregulatory models were based on measured values [7,25], which allowed us to examine the temperature elevation for local RF exposure in rats [7,25] and rabbit eyes [6,55,56] with good accuracy. However, extrapolation of these results from animals to humans is considered conservative [2]; lower temperature elevation may be expected in actual human tissues.

The antenna position was chosen to be different from the how people actually use mobile phones, because we decided to set the antenna position for accurate comparison of the thermal response between human and rat models. In that situation, the antenna is likely to be present near pinna, cheek, and mouth. For a dipole antenna located at the side of the pinna, most of the power is absorbed in the pinna [57]. For a dipole antenna located close to the cheek or mouth, the temperature elevation in the brain obviously decreases because of distance.

The thermal damage depends on tissue sensitivity, temperature, and exposure time [24]. The cumulative equivalent minutes at 43 °C are used as a model to calculate the thermal dose. In this evaluation, the thermal time constant is essential. Focusing on the brain, which is a highly heat-sensitive tissue, the thermal time constant in humans was more than twice that in rats. Thus, the exposure time required for thermal damage is correspondingly increased. Furthermore, because the characteristics of the temperature rise of deep tissues in rats and humans are different, extrapolation from small animals to humans in deep tissues needs further attention. However, these findings suggest that excessive temperature elevation is caused with increasing frequency in rat brains, but only marginally occurs in the human brain. These results exclude the possibility of thermally induced brain tissue damage for the limits stated in current international guidelines, especially at higher frequencies. To determine exactly how conservative these limits are when applied to humans, further experimental studies are necessary.

The thermal time constant obtained here would also be useful to interpret the experimental data discussing the thermal and non-thermal effects of RF fields, because the temperature elevation may not always be monitored in the measurements [58,59,60].

## 5. Conclusions

This study investigated the differences in the temperature elevation and thermal time constant between rat and human models exposed to dipole antennas at frequencies of 3–10 GHz. We computationally estimated the time course of temperature elevation by considering the vasodilation. It should be noted that the temperature elevation and time course are quite different between rat and human models, especially in deep tissue such as the brain. The characteristics of temperature elevation vary in humans owing to the shape and size of the head and distance to the antenna. We then proposed the effective SAR volume as a metric to estimate the thermal time constant, indicating that there is a good correlation between them, even if head shapes are significantly different, such as in rats and humans. These findings should be useful for extrapolating small animal studies to humans.

## Figures and Tables

**Figure A1:**
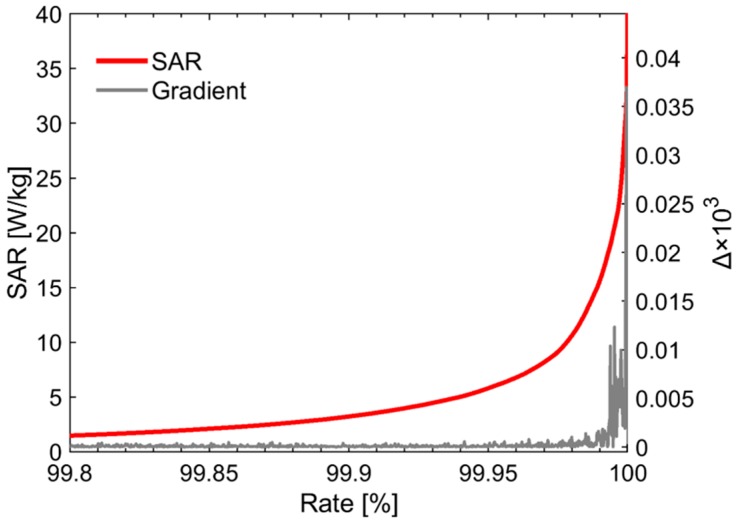
Computed results of SAR and its gradient for voxels of which the SAR has the highest value of 0.2% in the TARO head model.

## Appendix A. Comment of Maximum SAR

Section 2.2.5 introduces the effective exposure volume, which is a volume larger than *SAR_max_*/*e*, as an assessment of the extent of the SAR pattern. One of the disadvantages of using voxelized anatomical human models is that the computed electromagnetic absorbed power suffers from computational artifacts, especially around the model surface. This error is notable at low frequencies [61], but may still occur in computation at high frequencies. We defined the voxel *SAR_n_* as the *n*^th^ element of the list of SAR values sorted in ascending order. The gradient Δ was computed for each *SAR_n_* by the following equation:

(A1) $$\Delta_{n}=\frac{SAR_{n+1}-SAR_{n}}{\left(SAR_{n}+SAR_{n+1}\right)/2},$$

The first significantly different value of the gradient is defined as the detection point of the outlier [62]. Figure A1 shows the voxel values corresponding to an SAR with the highest value of 0.2%, and the gradient in the TARO model. As shown in Figure A1, the gradient significantly increased above 99.99% owing to the effect of calculation using a voxelized model. The gradient of the SAR value in the other head models also significantly increased above 99.97–99.99%. *SAR_max_* was the value obtained by removing the highest value of 0.01%. The removed volumes were 0.4–0.5 cm^3^ and 0.02 cm^3^, which corresponded to 0.5–0.8 g and 0.04 g in human and rat models, respectively. The removed volumes are generally located in complex shapes, such as around pinna. Note that an extensive analysis was conducted for low-frequency exposure where in situ electric fields were evaluated in voxelized models [62]. Similarly, the metric for evaluating SAR should be averaged over 10 g of tissue, and thus is not discussed extensively for RF exposures. The main purpose of this metric is to evaluate the SAR volume appropriately rather than for defining the physical meaning of the temperature.
